# Carriage of *Staphylococcus aureus* in Thika Level 5 Hospital, Kenya: a cross-sectional study

**DOI:** 10.1186/2047-2994-3-22

**Published:** 2014-07-15

**Authors:** Alexander M Aiken, Irene M Mutuku, Artur J Sabat, Viktoria Akkerboom, Jonah Mwangi, J Anthony G Scott, Susan C Morpeth, Alexander W Friedrich, Hajo Grundmann

**Affiliations:** 1 London School of Hygiene and Tropical Medicine, Keppel Street, London, UK; 2 Kenya Medical Research Institute-Wellcome Trust Research Programme, Kilifi, Kenya; 3 Department of Medical Microbiology, University of Groningen, University Medical Center Groningen, Groningen, The Netherlands; 4 Thika Level 5 Hospital, Thika, Kenya

**Keywords:** *Staphylococcus aureus*, MRSA, Kenya, Hospitals, Carriage prevalence

## Abstract

**Background:**

Methicillin-resistant *Staphylococcus aureus* (MRSA) is an important nosocomial pathogen but little is known about its circulation in hospitals in developing countries. We aimed to describe carriage of *S.aureus* amongst inpatients in a mid-sized Kenyan government hospital.

**Methods:**

We determined the frequency of *S.aureus* and MRSA carriage amongst inpatients in Thika Hospital, Kenya by means of repeated cross-sectional ward surveys. For all *S.aureus* isolates, we performed antibiotic susceptibility tests, genomic profiling using a DNA microarray and *spa* typing and MLST.

**Results:**

In this typical mid-sized Kenyan Government hospital, we performed 950 screens for current carriage of *S.aureus* amongst inpatients over a four month period. We detected *S.aureus* carriage (either MSSA or MRSA) in 8.9% (85/950; 95%CI 7.1-10.8) of inpatient screens, but patients with multiple screens were more likely have detection of carriage. MRSA carriage was rare amongst *S.aureus* strains carried by hospital inpatients – only 7.0% (6/86; 95%CI 1.5-12.5%) of all isolates were MRSA. Most MRSA (5/6) were obtained from burns patients with prolonged admissions, who only represented a small proportion of the inpatient population. All MRSA strains were of the same clone (MLST ST239; *spa* type t037) with concurrent resistance to multiple antibiotic classes. MSSA isolates were diverse and rarely expressed antibiotic resistance except against benzyl-penicillin and co-trimoxazole.

**Conclusions:**

Although carriage rates for *S.aureus* and the MRSA prevalence in this Kenyan hospital were both low, burns patient were identified as a high risk group for carriage. The high frequency of genetically indistinguishable isolates suggests that there was local transmission of both MRSA and MSSA.

## Background

*Staphylococcus aureus* is both a human commensal and an important pathogen [1]. Methicillin-resistant *S.aureus* (MRSA) is a common cause of healthcare-associated infections in both developed and developing countries, though limited information is available from the latter [2]. In hospital wards in developing countries, the risk of nosocomial transmission is likely to be high due to close physical proximity of patients, inadequate staffing, unreliable water-supply for handwashing, lack of alcohol hand-rub, isolation facilities or expertise for infection control [3] - these could represent near-ideal conditions for nosocomial circulation of drug-resistant bacteria. In sub-Saharan Africa, screening for MRSA carriage during hospital admission is rarely considered a healthcare priority and is infrequently performed, so patients carrying MRSA in hospitals in this region are unlikely to be identified or isolated.

Carriage of *S.aureus* is most commonly detected in the anterior nares (nostrils), though other sites on the body are frequently colonised, most commonly the groin and the axillae [1]. Isolates from the nares are normally indistinguishable by molecular typing from those found on other body sites [4]. Most *S.aureus* infections originate from self-carried strains, although disease only develops in a small minority of carriers [4,5]. Cross-sectional studies in adult populations have typically found nasal carriage rates of around 25%, although much of this work has been conducted in high-income settings [1]. Carriage of *S.aureus* (either MRSA or methicillin-sensitive *S.aureus* (MSSA)) often lasts from months to years, and tends to be longer amongst individuals with chronic skin disease [6].

MRSA expresses resistance to all β-lactam antibiotics (penicillins, cephalosporins, carbapenems) by producing a penicillin-binding protein (PBP2a) from the *mecA* gene in the staphylococcal cassette chromosome (SCC*mec*). MRSA was first described in 1961 in the UK, very soon after the introduction of anti-staphylococcal penicillins [7]. Some of the earliest reports of MRSA in sub-Saharan African came from various Kenyan Hospitals (including Thika Hospital) in the early 1990’s, where it was principally found amongst burns patients [8]. The prevalence of MRSA is the percentage of a collection of *S.aureus* isolates expressing resistance to methicillin – a marker for the presence of *mecA*. This has been estimated in a small number of studies in sub-Saharan Africa over the past 25 years, mostly based on clinical (disease-causing) isolates obtained at university teaching hospitals in major cities [9-12]. However, there are relatively few studies examining the prevalence of *S.aureus* carriage and of MRSA amongst these carriage isolates from patient populations in sub-Saharan Africa [13-18] – see Table 1. These studies show considerable variation in MRSA carriage prevalence amongst inpatient groups: from as high as 21% amongst TB inpatients in Tulega Ferry, South Africa to as low as 1.3% amongst a population comprising paediatric and surgical inpatients in Accra, Ghana - it is unclear what gives rise to this diversity.

**Table 1:** **Estimates of carriage of *S.aureus *** **and MRSA in patient populations in sub-Saharan Africa**

**Study location**	**Carriage isolates from**	**Proportion with *S.aureus *** **carriage**	***S.aureus *** **carriage rate**	**Proportion of MRSA/*S.aureus***	**MRSA**	**Overall MRSA carriage rate**
Mogadishu, Somalia [13]	Paediatric inpatients	21/46	46%	1/21	5%	2.2%
Tulega Ferry, South Africa [14]	Inpatients with TB	13/52	25%	11/13	85%	21%
Lagos, Nigeria [15]	HIV + patients (outpatients)	124/374	33%	20/124	16%	5.3%
Lambaréné, Gabon [16]	Sickle-cell disease paediatric outpatients	34/73	47%	1/34	2.9%	1.4%
Ife-Ife, Nigeria [17]	Patients on admission to surgical wards	61/192	32%	7/61	11%	3.6%
Accra, Ghana [18]	Inpatients (Paediatric and Surgical Departments)	63/452	14%	6/63	10%	1.3%

Most MRSA strains in Africa appear to derive from a small number of common ancestors: a recent study examining 86 MRSA isolates from clinical isolates at five hospitals in West Africa and Madagascar found that 88% of these isolates were from one of three clonal strains [12]. By contrast, there was wide diversity amongst MSSA clones from the same institutions [11]. No similar studies have been reported from East Africa and the types of MRSA circulating in East Africa are largely unknown.

We aimed to describe the prevalence and diversity of MSSA and MRSA carriage amongst inpatients in a typical mid-sized public-service hospital in Kenya.

## Methods

### Study setting and patient procedures

Thika Level 5 Hospital is a 300-bed Government Hospital in the town of Thika, approximately 50 km north-east of Nairobi, in Central Province, Kenya. It provides medical, surgical, gynaecological, obstetric and paediatric services to a mixed urban and rural population and has a total of seven inpatient wards. Throughout this study there was no full-time infection control nurse in Thika Hospital, though a Hospital Infection Prevention and Control Committee met monthly. Daily bed occupancy rates in adult wards in 2011 were typically between 120 and 140% and all patients were nursed in communal bays.

Between the 11^th^ July and 7^th^ November 2011, each week all current inpatients in two out of the five adult inpatient wards (rotating between male and female medical, male and female surgical and gynaecological) were screened for carriage of *S.aureus.* Any patients who stayed in the ward overnight prior to screening were eligible for inclusion, provided that they (or a family member) were able to give consent. The surgical wards included some paediatric patients receiving treatment for surgical conditions, who were also included in screening. Patients who were MRSA-negative were repeatedly screened throughout their entire period of hospitalisation, resulting in some patients being screened multiple times. Initial results regarding MRSA carriage were relayed to patients and clinicians as soon as these became available. Patients found to have carriage of MRSA were subsequently treated with vancomycin if an infection with *S.aureus* was suspected. Patient data were collected from patient notes or by direct interview during screening and single-entered into a local database. Statistical analysis was carried out using STATA v12.

### Ethics statement

This study was approved by the Kenya Medical Research Institute National Ethical Review Committee. All patients (or their relatives) gave written informed consent to nasal and skin swab collection.

### Swab processing

Paired nasal and axillary skin swabs were collected from each patient and were immediately transferred to Mannitol Salt broth (HiMedia Laboratories, Mumbai, India) for overnight selective enrichment. The following day, aliquots were plated onto Blood Agar (BA) media. Single colonies from plates which showed suspected *S.aureus* colony growth, were further processed by Gram stain, catalase and coagulase test, and checked for the ability to hydrolyse DNA. Disc diffusion tests with cefoxitin (10 μg) were used to identify suspected MRSA according to British Society for Antimicrobial Chemotherapy guidelines. All media were locally prepared and batch controlled using appropriate internal quality control strains (*S.aureus* ATCC 25923, *S.epidermidis* ATCC 12228, *E.coli* ATCC 25922). These initial screening tests were performed in Thika Hospital, Kenya. Isolates were initially kept at -20°C and then transferred to -80°C for storage. Isolates were later shipped to the University Medical Centre Groningen, the Netherlands for further investigations.

### Antimicrobial susceptibility testing

Susceptibility to a standard panel of antibiotics plus chloramphenicol was performed using the automated Vitek 2 system (bioMerieux, Marcy l’Etoile, France). The results were interpreted in accordance with the 2012 guidelines of the Clinical and Laboratory Standards Institute. Isolates positive for inducible clindamycin resistance were assumed to be resistant to lincosamines and macrolides.

### Extraction of genomic DNA

Total DNA was prepared from a loop of *S.aureus* cells lifted from blood agar plates and transferred to 2-ml tubes containing 500 μl of water and zirconia/silica beads. The tubes were fixed in a TissueLyser homogenizer (Qiagen, Venlo, Netherlands) and the cells were disrupted by vortexing for 5 min at 30 Hz. The suspensions were clarified by centrifugation (14,000 rpm for 10 min), and the supernatant was used for DNA extraction with the DNeasy Blood & Tissue kit (Qiagen) according to the manufacturer’s protocol. The DNA concentration was quantified using a NanoDrop spectrophotometer (NanoDrop Technologies, Wilmington, DE) at 260 nm.

### Genotyping

*Spa* typing [19] and Multi Locus Sequence Typing (MLST) [20] were performed as described previously. *Sp*a types were assigned by Ridom StaphType software version 1.4.6 (Ridom GmbH, Würzburg, Germany) and the Ridom SpaServer (http://www.spaserver.ridom.de) [21] after adhering to the SeqNet.org quality procedure [22]. A single representative of each *spa* type was analyzed by MLST. Sequences of each MLST locus were compared to the data in the *S.aureus* MLST database (http://saureus.mlst.net/), and resulting allelic profiles were assigned to particular sequence types (STs) for each isolate. eBURST software (v3, http://eburst.mlst.net) was used to classify related Sequence Types (STs) into clonal complexes (CCs) A singleton was defined as a sequence type that did not group with any clonal complex. The diagnostic DNA microarrays (StaphyType; Alere Technologies, Jena, Germany) and StaphyType DNA microarray kit were used for study of the presence of genes encoding species markers, agr types and virulence factors as described previously [23].

## Results

### Carriage of *S.aureus*

A total of 950 screening swabs were obtained from 733 inpatients in the medical, surgical and gynaecological wards of Thika Hospital. Patients ranged in age between 1 and 90 years and 52% of screens were performed in male patients (see Table 2). We found no association between carriage of *S.aureus* and either age-group or sex (χ^2^ test; p > 0.1). As a result of the repeated rounds of cross-sectional inpatient screening, an average of 1.4 screens were performed per patient over the study period (range 1–8 screens; see Table 3).

**Table 2:** Patient characteristics in screening tests

**Characteristic**	**Screening negative (%)**	**Any *S.aureus *** **detected (%)**	**MRSA detected (%)**
Median age, years (range)	34 (1–90)	40 (2–80)	27 (2–57)
Age < 14 yrs	103 (12)	4 (5)	4 (67)
Male sex	450 (52)	48 (56)	4 (67)
Ward location			
Surgical - Male	298 (52)	44 (34)	4 (67)
Surgical - Female	215 (25)	22 (26)	2 (33)
Medical - Male	127 (15)	7 (8)	0 (0)
Medical - Female	141 (16)	8 (9)	0 (0)
Gynaecology	84 (10)	4 (5)	0 (0)
**Total**	**865 (100)**	**85 (100)**	**6 (100)**

**Table 3:** **Rounds of *S.aureus *** **screening amongst inpatients**

**Number of screens performed**	**Number of patients**	**Total number of screens**	***S.aureus *** **not detected on screen**	**Screens with MSSA detected**	**Screens with MRSA detected**	***S.aureus *** **ever detected in patient (%)**
1	609	609	566	39	4	43/609 (7.1%)
2	72	144	125	18	1	14/72 (19.4%)
3	27	81	74	7	0	5/27 (18.5%)
4	15	60	50	10	0	7/15 (46.7%)
5	7	35	33	2	0	2/7 (28.6%)
6	1	6	5	1	0	1/1 (100%)
7	1	7	4	3	0	1/1 (100%)
8	1	8	7	0	1	1/1 (100%)
**Total**	**733**	**950**	**865**	**79**	**6**	**74/733 (10.1%)**

A total of 85 inpatient screenings (85/950; 8.9%; 95%CI 7.1-10.8) detected carriage of *S.aureus*. One patient had two distinct *S.aureus* strains simultaneously (one MRSA and one MSSA). Thus, a total of 86 *S.aureus* isolates were collected. Species identification was confirmed by the presence of the *S.aureus*-specific genes (*spa*, *coA*, *nuc1*) and *mecA* for MRSA. Among the *S.aureus* isolates obtained, six (6/86; 7.0%; 95%CI 1.5-12.5%) were MRSA. All of these were obtained from surgical patients, comprising three adult men and one male child (male surgical ward) and one woman and one female child (female surgical ward). All of these patients had been hospitalised for a prolonged period, five with extensive burns and one with a fractured femur. The median admission length prior to MRSA detection was 27 days (range 25–172 days). When comparing patients with single versus multiple rounds of screening performed, there was evidence that having multiple screens was associated with higher likelihood of *S.aureus* being detected (χ^2^ test; p < 0.001 – see Table 3). Out of all patients in the study, a total of 74 were found to have carriage of *S.aureus* in at least one screen (74/733; 10.1%; 95%CI 7.2-12.3).

### Antibiotic resistance

All six MRSA isolates were co-resistant to gentamicin, ciprofloxacin, tetracycline and co-trimoxazole and five were also resistant to lincosamine and macrolide antibiotics. All MRSA isolates were susceptible to chloramphenicol, vancomycin and mupirocin. Amongst the MSSA isolates obtained, resistance to penicillin, tetracycline and SXT was common, but not to any other antibiotics – see Table 4.

**Table 4:** Sensitivity patterns of MRSA and MSSA carriage isolates from Thika Hospital, Kenya

**Antibiotic**	**Proportion susceptible (%)**	
	**MSSA (n = 80)**	**MRSA (n = 6)**
Cefoxitin	100	0
Benzylpenicillin	26	0
Co-trimoxazole	60	0
Tetracycline	85	0
Gentamicin	99	0
Ciprofloxacin	100	0
Erythromicin/Clindamycin	99	17
Rifampicin	99	83
Chloramphenicol	100	100
Vancomycin	100	100
Mupirocin	100	100

### Diversity of isolates established by *spa* typing and MLST analyses

The 86 *S.aureus* isolates were assigned to 28 *spa* types, ranging in length between four (t10499) and 12 (t005) repeats. Two new repeats, r558 and r559, and five new *spa* types, t10496, t10497, t10498, t10499 and t10960, were found and assigned over the course of this study. Sixteen *spa* types were represented by 2 or more isolates (73 isolates in total), while 13 *spa* types contained a single isolate. All six MRSA isolates identified were of *spa* type t037. Among the MSSA isolates, t223 (n = 15) was the most frequent *spa* type, followed by t064 (n = 10) and t131 (n = 10). Other *spa* types (n = 25) were represented by four or less MSSA isolates. Isolates obtained from initial patient-screens were less likely to have *spa* types with multiple (≥3) occurrences (39/57, 68%) than those isolates obtained from subsequent rounds of screening (24/29, 83%), though this difference did not reach statistical significance (χ^2^ test; p = 0.16).

Using MLST, twenty STs were identified. Five new MLST allelic profiles and three new STs were found; the latter were assigned as ST2429, ST2430, and ST2431. All six MRSA isolates belonged to international clone ST239. The correspondence between MLST and *spa* typing is shown in Table 5.

**Table 5:** **MLST and spa types from 86** ***S.aureus *** **isolates obtained at Thika Hospital, Kenya**

MLST CC*	**ST within CC (number of isolates)**	***spa *** **types (number of isolates, if >1 *spa *** **type)**
CC22	ST22 (17)	t223 (15), t005 (1), t10498 (1)
CC8	ST8 (14)	t064 (10), t121 (3), t10497 (1)
CC121	ST2430 (4)	t645
	ST121 (3)	t314
CC239	ST239 (6)	t037†
CC97	ST97 (5)	t359 (2), t1965 (2), t267 (1)
CC5	ST5 (3)	t002
CC30	ST30 (3)	t318
CC7	ST7 (3)	t091
CC6	ST6 (2)	t701
CC15	ST15 (2)	t084 (1), t491 (1)
CC25	ST25 (2)	t3772
CC72	ST72 (2)	t148 (1), t4353 (1)
CC1	ST1 (1)	t127
CC45	ST45 (1)	t015
Singletons	ST1290 (10)	t131
	ST152 (4)	t355
	ST2431 (2)	t10496
	ST2019 (1)	t10499
	ST2429 (1)	t10960

* = Clonal Complex nomenclature derived from MLST database.

† = all 6 isolates of t037 were MRSA, all other isolates were MSSA.

Using eBURSTv3, all MLST STs obtained in the study were assigned to CCs. This method identified ST2430 as a single locus variant of CC121; the remaining 18 STs were unrelated. Subsequently, MLST data obtained in this study were compared to the *S.aureus* MLST database. Of the 68 isolates which were grouped into 14 CCs, the majority of these (64/68, 94%) had a founder sequence type – see Table 5.

### Microarrays

The distribution of genes of the core variable genome and the identification of the allelic variants of genes of the core genome varied between MLST sequence type. Amongst the 6 MRSA isolates, all carried the SCCmec III element, but none carried the lukF/lukS (PVL) genes. Amongst the 80 MSSA isolates, the frequency of carriage of the following genetic elements was as follows: *lukF-PV/lukS-PV* (PVL; n = 15; 19%), toxic shock syndrome toxin 1 (*tst1*; n = 18; 23%), staphylococcal enterotoxin a (*sea*, n = 17; 21%) and exfoliative toxin a (*eta*; n = 6; 8%). Full details of the microarray results including descriptions of profiles for all isolates are given as supplementary material in Additional file 1.

## Discussion

In this typical mid-sized Kenyan government hospital, carriage of *S.aureus* amongst inpatients was found to be relatively infrequent (8.9%; 95%CI 7.1-10.8) in individual screens. Due to the repeating cross-sectional sampling design, this result may underestimate the actual inpatient prevalence due to a time-dependant bias [24] introduced by only performing multiple rounds of screening in patients with longer admissions. The actual prevalence might be better estimated by the proportion of patients in the study who were ever detected to have *S.aureus* carriage (74/733; 10.1%; 95%CI 7.2-12.3) – though some of these patients probably acquired carriage during admission. MRSA constituted only 6.9% (95%CI 1.5-12.5%) of all *S.aureus* isolates obtained. Both the overall carriage rate and the proportion of MRSA were surprisingly low, given that nasal carriage of *S.aureus* in European populations is typically around 25% [1,25] and the crowded conditions in Thika Hospital should have led to frequent transmission of MRSA. The small number of MRSA isolates obtained leaves us with a wide confidence interval for the true microbiological prevalence.

Almost all MRSA carriage was confined to patients with burns who constituted approximately 10% of adult inpatients admitted to the surgical wards and approximately 5% of inpatients overall. In sub-Saharan Africa, prolonged hospital admission after burns injuries are common [26], and as specialist burns units are rarely available, these patients often make up a sizeable proportion of the general inpatient population. Burns patients are at increased risk of invasive bacterial disease [27] and hence colonisation of these patients with a multi-drug resistance organism is of particular concern. Chloramphenicol is a widely used 2nd line antibiotic in sub-Saharan Africa and as all isolates in Thika Hospital were found to be susceptible to this drug, this might be a suitable empirical alternative to vancomycin for suspected MRSA infections in this setting.

These Thika MRSA isolates belong to the widespread ST239 strain, formerly known as the Hungarian/Brazilian epidemic strain which is typically associated with hospital-acquisition. This has been documented to occur in sub-Saharan Africa in Senegal, Niger [12], South Africa [28] and was noted to be common amongst hospitalised burns patients in Nigeria [10]. The pattern of multi-class antibiotic resistance that we found in Thika Hospital is typical of this strain in African settings [12].

When designing this study, we expected *S.aureus* and MRSA to occur considerably more frequently than we actually found. We used a repeated cross-sectional approach for detecting carriage but this may have missed some cases of nosocomial acquisition occurring late in admission, which could have been detected by additional screening on discharge.

The reason for the surprisingly low overall rate of *S.aureus* carriage (8.9%) in our study is unclear. We feel that is it unlikely that procedural errors with swab collection could account for this – all swab collection was supervised in person and regularly quality-controlled by one investigator (AA) and an experienced researcher (HG) made an on-site review of the collection and processing methods in the first month of the study. The anterior nares and axillae are widely accepted to be reliable sites for *S.aureus* detection [1] and we used an enrichment culture intermediate step to assist with detection of small numbers of organisms. However, we accept that swabs from the skin surface have inherently limited sensitivity to detect carriage organisms and we note that in our study, patients who had multiple screenings performed were more likely to ever have had *S.aureus* carriage detected. It is therefore possible that a single round of screening is not sufficient to exclude carriage in this setting. Alternatively, progressive acquisition of carriage during hospital admission, as evidenced by our molecular studies, could provide some explanation for this finding of increasing carriage with repeated screening. Genetic differences might also provide an explanation for a low carriage rate – human genetic factors are thought to be important determinants for persistent colonisation with *S.aureus*[29].

There were some intriguing findings revealed by the molecular analyses. In particular, we note that in comparison to isolate collections from other settings, there were high proportions of isolates with genes for the Panton-Valentine Leukocidin (PVL, 15/86, 19%) and toxic shock syndrome toxin 1 (*tst1*, 18/86, 23%). From the perspective of staphylococcal classification systems, many of the *spa* types (18%; 5/28) and MLST profiles (15%, 3/20) identified in this population were novel. This is reflective of the limited contribution of African sites to the relevant typing databases. The lack of diversity amongst the MRSA and MSSA isolates obtained in our institution over a four month period suggests that these were probably being transmitted by local (within-hospital) routes rather than repeated introduction from external sources, though without greater knowledge of the diversity of strains circulating in Kenya or East Africa as a whole, we cannot be certain of this. Due to overcrowding and lack of isolation facilities, eliminating nosocomial transmission of organisms such as MRSA in institutions like Thika Hospital will be challenging.

Antibiotic resistance is a growing concern in developing countries [30] and local studies are needed to understand the transmission of drug-resistant organisms and guide strategies for containment. MRSA is an important nosocomial pathogen in developed countries but is little-known in sub-Saharan Africa. This is the largest study examining inpatient carriage of *S.aureus* in sub-Saharan Africa and it was conducted in a typical mid-sized Kenyan Government Hospital. We found inpatient carriage of MRSA to be relatively infrequent and was mainly confined to patients with burns. The lack of genetic diversity amongst the isolates obtained suggests “within-hospital” transmission. The multiple drug resistance of MRSA found in Thika hospital coupled with the vulnerability of burns patients to invasive bacterial infection is a cause for concern. Thika Hospital faces many infection control challenges that are typical of those encountered in African healthcare facilities. Much work is needed in Kenya and throughout the region to formulate appropriate national and local policies to tackle issues such as MRSA and, more importantly, to put these into effective practice.

## Competing interest

All authors declare that they have no competing interest.

## Authors’ contributions

AA, JAGS and HG designed the study. AA, JM and HG supervised collection of samples from patients. IMM, SCM, AJS, VA and AWF conducted and supervised laboratory analysis of samples in Thika Hospital and UMCG. AA drafted the manuscript and all authors read and approved the final manuscript.

## Supplementary Material

Additional file 1 **Full *S.aureus *** **microarray results from Thika Hospital.** Click here for file
