# Supplementary material for: Carriage of Staphylococcus aureus in Thika Level 5 Hospital, Kenya: a cross-sectional study
Source: Antimicrob Resist Infect Control. 2014 Jul 15;3:22. doi: 10.1186/2047-2994-3-22 (PMC4107749; doi:10.1186/2047-2994-3-22)
Supplement: Additional file 1 — Full S.aureus microarray results from Thika Hospital. [file 2047-2994-3-22-S1.docx]

Additional file 1

This Additional file describes the full microarray results from the *S.aureus* isolates from Thika Hospital.

Factors present in all isolates were as follows: virulence genes hld, sspA and sspB encoding hemolysin delta, serine protease V8 and cysteine protease, respectively; immune evasion gene isaB encoding immunodominant antigen B; adhesion factors genes clfA, clfB, eno, ebpS and vwb encoding clumping factor A, clumping factor B, enolase, cell wall associated fibronectin-binding protein and von Willebrand factor, respectively; the intercellular adhesion locus (*ica*) responsible for biofilm production, however only the icaA gene from this locus was present in all isolates.

ST239-MRSA

All six MRSA isolates carried the SCCmec III element, the mercury resistance operon, ccrC, the virulence genes sek and seq, the protease splA, splB and splE genes but phage-associated genes sak, scn and chp were present only in four isolates. Five isolates carried the haemolysins genes, *hla* and *hlb*. All isolates had the same set of the MSCRAMM genes (*bbp*, *cna*, *ebh*, *fib*, *fnbA*, *fnbB*, *map*, *sdrC*, *sdrD*, and *sasG*). No MRSA isolates carried the lukF/lukS (PVL) genes.

ST22-MSSA

All isolates carried the enterotoxin gene cluster *egc* (*seg*, *sei*, *sem*, *sen*, *seo*, and *seu*) and almost all, with the exception of a single isolate with the spa type t005, were positive for the gene encoding the toxic shock syndrome toxin (*tst1*). All isolates were characterized by the lack of the protease genes *splA*, *splB* and *splE* but contained β-haemolysin-converting phages (sak, chp and scn), however, in two isolates only *sak* was detected. The *hla* and *hlb* genes encoding alpha- and beta-hemolysins, respectively, were found in all isolates with the exception of a single isolate in which the *hla* gene was absent. In all isolates the MSCRAMMs genes such as *cna*, *fib*, *sdrC* and *sasG* were present, while carriage of the *bbp*, *fnbA*, *fnbB*, *map*, and *sdrD* genes was variable.

ST8-MSSA

The majority of isolates (12 out of 14) from this group possessed the enterotoxin profile (*sea*+seb+sek+seq) characteristic for the strain of CC8-MRSA-IV which is known as USA500. Moreover, one of the MSSA USA500 isolates possessed the *tst1* gene. Two remaining isolates from this group were equipped with the *sea* gene, and one of them additionally had the *sek* gene. All ST8-MSSA isolates from Thika Hospital showed homogenous distribution of the hemolysins genes (*hla* and *hlb*, with the exception of a single isolate in which the *hla* gene was not detected), immune evasion genes (*sak, scn, splA, splB,*and *splE*), and the MSCRAMMs genes (*bbp*, *ebh*, *fib*, *fnbA*, *fnbB*, *map*, *sdrC*, *sdrD*, and *sasG*).

ST1290-MSSA

The enterotoxin genes were not detected in this group but majority of the isolates (6 out of 10) harboured the exfoliative toxin gene eta. All isolates had either the hla or hlb gene. The immune evasion genes sak, scn, splA, splB, and splE were found in all isolates and a single isolate had additionally the chp gene. The MSCRAMMs profile *bbp*+*ebh*+*fib*+*fnbA*+*fnbB*+ *sdrC*+*sdrD*+*sasG* was found in all isolates, while the *map* gene was additionally found in seven isolates.

CC121-MSSA

All isolates carried the bi-component toxin Panton-Valentine leukocidin (PVL) genes and carried *seb*, *sec*, and the *egc* enterotoxin locus (*seg*, *sei*, *sem*, *sen*, *seo*, and *seu*). The *hla* and *hlb* genes coding for hemolysins alpha and beta were found in all isolates of this clonal complex. The genes associated with beta-haemolysin converting phages such as *sak* and *scn* were present, while *chp* was absent in all CC121 MSSA isolates. The distribution of the protease genes in this clonal complex was sequence type specific. The isolates of ST2430 possessed the *splA* and *splB* genes, whereas the isolates of ST121 had only the *splB* gene. All isolates had the MSCRAMMs genes as follows: *bbp*, *cna*, *ebh*, *fnbA*, *fnbB*, *map*, *sdrC*, and *sdrD*. Moreover, four out of seven isolates possessed additional adhesion gene encoding the *S.aureus* surface protein G (*SasG*), and this distribution was not ST specific.

ST97-MSSA

This group of isolates was characterized by the lack of the toxin genes. All other groups of genes were homogeneously distributed among the isolates: hemolysins (*hla* and *hlb*), immune evasion (*sak*, *scn*, *splA*, *splB*, and *splE*) and MSCRAMMs (*bbp*, *ebh*, *fib*, *fnbA*, *fnbB*, *map*, *sdrC*, *sdrD*, and *sasG*)

ST152-MSSA

All isolates were PVL-positive and enterotoxins-negative. The only toxin detected was *EdinB*: this structural gene was present among three out of four isolates. Two isolates possessed the *hla* and *hlb* genes, one had the *hla* gene and one isolate had neither *hla* nor *hlb*. The protease genes *splA*, *splB* and *splE* were not detected in all isolates. Two MSCRAMMs profiles, *bbp*+*can*+*ebh*+*fnbA*+*fnbB*+*sdrD* and *bbp*+*can*+*ebh*+*fnbA*+*fnbB*+*sdrD*+*sasG*, were equally found in ST152 MSSA isolates.

ST7-MSSA

The *sea* gene was detected in all three isolates from this group. Moreover, one of the ST7 isolates was positive for the *sed* gene. All isolates had the same hemolysin and immune evasion gene profiles (*hla*, *hlb* and *sak*, *scn*, *splA*, *splB*, *splE*, respectively). Almost all isolates had the same MSCRAMM gene profile (*bbp*+*ebh*+*fib*+*fnbA*+*fnbB*+*map*+*sdrC*+*sdrD*) and the only difference was found for a single isolate which possessed additionally the *sasG* gene.

ST5-MSSA

All three isolates shared the same hemolysin and immune evasion gene content (*hla*, *hlb*, *sak*, *chp*, *scn*, *splA*, and *splB*). Two isolates showed the same distribution of superantigens (*seb*, *sek*, *seq*, and the cluster *egc*) and MSCRAMMs (*bbp*, *ebh*, *fib*, *fnbA*, *fnbB*, *map*, *sdrC*, *sdrD*, and *sasG*). Third isolate harbored the enterotoxin gene cluster *egc* and almost the same MSCRAMM genes with the exception of *sdrD* which was not detected in the genome.

ST30-MSSA

All 3 isolates had the same content for genes encoding hemolysins (*hla* and *hlb*) immune evasion (*sak, chp, scn, splE*) and superantigens (*egc* cluster: *seg*, *sei*, *sem*, *sen*, *seo*, and *seu*). Moreover, all isolates were characterized by the presence of genes encoding PVL. The only difference between isolates was in the MSCRAMM content: two isolates possessed the *bbp*, *cna*, *ebh*, *fib*, *fnbA*, *fnbB*, *map*, *sdrC* and *sdrD* genes. The third isolate had almost the same MSCRAMM gene content as the two other isolates with the exception of the *fnbB* gene which was not detected in the genomic DNA.

ST2431-MSSA

Two isolates of this group did not have any toxin genes. They shared the same immune evasion genes, *sak* and *scn*, but differed in the content of the hemolysin (*hla*+/ *hlb*+ and *hla*+/ *hlb*-) and MSCRAMM (*bbp*, *ebh*, *fnbA*, *fnbB*, *sdrC*, *sdrD* and the same content plus *sasG*) genes.

ST6-MSSA

Both isolates shared exactly the same gene content. They showed the gene content as follows:

*hla*, *hlb*, s*ak, scn, splA, splB, splE, sea*, *bbp*, *cna*, *ebh*, *fib*, *fnbA*, *fnbB*, *map*, *sdrC*, *sdrD*, and *sasG*.

ST72-MSSA

This group was composed of two isolates with different *spa* types. The isolates shared the same hemolysin (*hla* and *hlb*), immune evasion (*sak,scn,splA,splB,splE*) and MSCRAMM (*bbp*, *ebh*, *fib*, *fnbA*, *fnbB*, *map*, *sdrC*, *sdrD*, and *sasG*) genes but differed substantially in the content of the superantigen genes. One of the isolates (with the *spa* type t148) harbored the enterotoxin *egc* genes, while second one (with the *spa* type t4353) possessed the *egc* cluster and also the *tst1*, *sec*, *sel* genes.

ST15-MSSA

Both isolates were characterized by the presence of the same gene content and they did not have any toxin genes. They yielded hybridisation signals for the hemolysin gene *hla*, immune evasion genes *chp*, *scn*, *splA*, *splB*, *splE*, and the MSCRAMM genes *bbp*, *ebh*, *fib*, *fnbA*, *fnbB*, *map*, *sdrC*, *sdrD*, *sasG*.

ST25-MSSA

Two isolates showed almost the same gene content: differences were only found for the hemolysin genes. One isolate had both the *hla* and *hlb* genes, while these genes were not detected in genomic DNA of second isolate. The immune evasion (*sak,chp,scn,splA,splB,splE*), superantigen (*sec*, *sel*, and the egc cluster) and MSCRAMM (*bbp*, *ebh*, *fib*, *fnbA*, *fnbB*, *map*, *sdrC*, *sdrD*) genes were the same in both isolates. The characteristic feature of the isolates from this group was the presence of the *etd* gene coding for exfoliative toxin D. This gene was not found in other isolates in this collection. Another exfoliative toxin gene detected in the ST25 isolates was *edinB*.

ST2429-MSSA

The one isolate of this group did not show any hybridization signal for the toxin genes. It revealed hybridization signals for: the *hla* gene; phage-associated genes sak, scn and chp; protease genes splA and splE; and the adhesion genes *cna*, *ebh*, *fib*, *fnbA*, *fnbB*, *sdrC* and *sasG*.

ST2019-MSSA

The one isolate did not have genes for enterotoxins and exfoliative toxins. However, the PVL genes were detected in its genomic DNA. The isolate possessed the *hla* and *hlb* genes for hemolysins alpha and beta, respectively. Immune evasion genes genes *chp* and *scn* were

present, whereas *sak* and protease *splA*, *splB* and *splE* genes were absent. The MSCRAMM genes *bbp*, *ebh*, *fnbA*, *fnbB*, *sdrC*, and *sdrD* were detected in this isolate.

ST1-MSSA

Genomic content of the MSSA ST1 representative showed the presence of the superantigen *sea*, *seh*, *sek*, and *seq* genes. The hybridization results also revealed that this isolate harbored the PVL genes, the hemolysin genes *hla* and *hlb*, immune evasion genes *sak*, *scn*, *splA*, *splB*, *splE*, and the MSCRAMM genes *bbp*, *cna*, *ebh*, *fib*, *fnbA*, *fnbB*, *sdrC*, *sdrD*, *sasG*.

ST45-MSSA

The only isolate of this group possessed the genes encoding enterotoxins *seb* and that of the *egc* cluster (*seg*, *sei*, *sem*, *sen*, *seo*, and *seu*). Moreover, it revealed hybridization signals for the hemolysins alpha and beta genes (*hla* and *hlb*) and phage-associated genes sak, scn and chp. Protease genes splA, splB and splE were not detected. The adhesion genes detected in the genomic DNA were as follow: *bbp*, *cna*, *ebh*, *fib*, *fnbA*, *fnbB*, *map*, *sdrC* and *sdrD*.
